# Glycyrrhetinic Acid Antagonizes Pressure-Induced Venous Remodeling in Mice

**DOI:** 10.3389/fphys.2018.00320

**Published:** 2018-04-04

**Authors:** Hanna Kuk, Caroline Arnold, Andreas H. Wagner, Markus Hecker, Carsten Sticht, Thomas Korff

**Affiliations:** ^1^Division of Cardiovascular Physiology, Institute of Physiology and Pathophysiology, Heidelberg University, Heidelberg, Germany; ^2^Medical Clinic V, University Hospital Mannheim, Heidelberg University, Heidelberg, Germany; ^3^Division of Cardiovascular Physiology, Institute of Physiology and Pathophysiology, Heidelberg University, Heidelberg, Germany; ^4^Medical Faculty Mannheim, European Center for Angioscience, Heidelberg University, Heidelberg, Germany

**Keywords:** spider veins, wall stress, venous hypertension, endothelial cells, venous remodeling

## Abstract

Development of spider veins is caused by the remodeling of veins located in the upper dermis and promoted by risk factors such as obesity or pregnancy that chronically increase venous pressure. We have repeatedly shown that the pressure-induced increase in biomechanical wall stress is sufficient to evoke the formation of enlarged corkscrew-like superficial veins in mice. Subsequent experimental approaches revealed that interference with endothelial- and/or smooth muscle cell (SMC) activation counteracts this remodeling process. Here, we investigate whether the herbal agent glycyrrhetinic acid (GA) is a suitable candidate for that purpose given its anti-proliferative as well as anti-oxidative properties. While basic abilities of cultured venous SMCs such as migration and proliferation were not influenced by GA, it inhibited proliferation but not angiogenic sprouting of human venous endothelial cells (ECs). Further analyses of biomechanically stimulated ECs revealed that GA inhibits the DNA binding capacity of the mechanosensitive transcription factor activator protein-1 (AP-1) which, however, had only a minor impact on the endothelial transcriptome. Nevertheless, by decreasing gelatinase activity in ECs or mouse veins exposed to biomechanical stress, GA diminished a crucial cellular response in the context of venous remodeling. In line with the observed inhibitory effects, local transdermal application of GA attenuated pressure-mediated enlargement of veins in the mouse auricle. In summary, our data identifies GA as an inhibitor of EC proliferation, gelatinase activity and venous remodeling. It may thus have the capacity to attenuate spider vein formation and remodeling in humans.

## Introduction

Pathologic remodeling of veins often results in significant morbidity and discomfort for the patient while remodeling of smaller, i.e., superficial veins results in formation of spider veins or telangiectasias that often cause a cosmetic nuisance/discontent. Spider veins comprise dilated, intradermal vessels less than 1 mm in diameter (venous telangiectasias) or between 1 and 3 mm in diameter (reticular veins) and often resemble varicosities albeit on a much smaller scale (Partsch, 2009; Smith, 2015). According to the clinical, etiologic, anatomic, and pathophysiological (CEAP) classification system of chronic venous diseases, telangiectasias/reticular veins are classified as C1 (Eklof et al., 2004).

A number of risk factors have been associated with the development of spider veins, including prolonged sitting or standing, pregnancy, being overweight, wearing tight undergarments or clothes which suggest an underlying increase in the hydrostatic venous filling pressure associated with the deep veins in the lower extremities (Sisto et al., 1995; Pfisterer et al., 2014a). While details of the pathophysiology of spider veins are still unknown, former work of our group revealed that chronic elevation of the venous pressure is sufficient to drive venous remodeling in mice (Feldner et al., 2011; Pfisterer et al., 2014a; Kuk et al., 2017). In fact, some reports hypothesize that telangiectasias may arise from varicose veins of larger vessels due to venous hypertension caused by valvular damage resulting in reflux and venous dilatation (Thomson, 2016). With reference to the law of Laplace, elevated pressure levels result in an increase in wall stress or biomechanical (over)load of the venous vessel wall within the affected venous network to drive its further remodeling (Partsch, 2009; Atta, 2012). When spreading to superficial veins, venous hypertension may lead to the activation of venous endothelial (EC) and smooth muscle (SMC) cells and may thus pose as a relevant determinant of spider vein formation. Therefore, counteracting mechanisms which are rate limiting for this process such as activation of the mechanoresponsive transcription factor activator protein 1 (AP-1) or cellular proliferation have been shown to interfere with venous remodeling (Feldner et al., 2011; Pfisterer et al., 2014b).

Here, we attempted to attenuate activation of venous endothelial and smooth muscle cells (SMCs) and enlargement of superficial veins by utilizing bioactive compounds frequently utilized in skin care products. From among them, we selected glycyrrhetinic acid (GA)—the predominant lipophilic bioactive compound extracted from the root of licorice—due to its broad range of beneficial properties comprising anti-inflammatory, anti-oxidative and anti-allergic effects(Chang et al., 2010; Jayasooriya et al., 2014; Kong et al., 2015). Moreover, it also bears some anti-proliferative features (Huang et al., 2014; Tang et al., 2015; Zhu et al., 2015) which makes it a prime candidate for interfering with cellular characteristics associated with venous remodeling. In view of the above, we tested the impact of GA on responses of biomechanically stressed venous endothelial cells (ECs) and/or SMCs *in vitro* and *in vivo*.

## Materials and methods

### Antibodies and reagents

The anti-Ki67 antibody was obtained from abcam (Cambridge, UK; ab16667). The DQ-gelatin (EnzChek gelatinase assay kit) was obtained from ThermoFisher Scientific (Pittsburgh, PA, USA: E12055).

### HUVEC/HUVSMC cell culture

Isolation of human umbilical vein endothelial and SMCs (HUVEC/HUVSMC) was approved from the Local Ethical Committee (document number 336/2005, Heidelberg Germany) and conformed to the principles outlined in the Declaration of World Medical Association declaration of Helsinki (1997). Parental consent was obtained for isolation of cells from the umbilical cords of the newborns.

HUVEC: Umbilical veins were flushed with Hank's buffer solution to remove residual blood. The veins were filled with 10 ml of dispase solution (3.1 g/l) and incubated for 30 min at 37°C. Veins were then flushed with 40 ml of M199 medium resulting in a cell-containing media suspension. Both M199 and dispase solutions were centrifuged at 160 × g for 5 min and the cell pellet was re-suspended in M199 media (Sigma-Aldrich, Germany) supplemented with EC growth supplement (PromoCell, Germany, C-39215), 5% FBS, 50 U/ml penicillin, 50 μg/ml streptomycin and 0.25 μg/ml Fungizone® antimycotic. The EC phenotype of these cells was confirmed by positive immunofluorescence for CD31 endothelial marker and assessment of a cobble-stone like morphology. The cells were routinely cultured on standard plates pre-coated with 2% (w/v) gelatin at 37°C, 5% CO_2_. Only cells subcultured up to passage 4 were utilized for all subsequent experiments.

HUVSMC: The umbilical veins were flushed with D-PBS buffer to remove residual blood. The tunica intima, the inner most layer of the vein was denuded of the ECs and the tunica media layer was cut into small pieces (~0.3 mm × 0.3 mm) which were spread around in a 6 cm cell culture Petri dish. The venous fragments were covered gently with 15% FCS, DMEM media making sure not to dislodge the attached vein segments. Approximately 2 weeks later the cell outgrowths were trypsinized and centrifuged for 5 min at 160 × g. The pellet was re-suspened in 15% FCS, DMEM media supplemented with 50 U/ml penicillin, 50 μg/ml streptomycin and 0.25 μg/ml Fungizone® antimycotic mix and transferred to T-75 cell culture flask. The cells were routinely cultured on standard tissue culture plates at 37°C, 5% CO_2_ and cells cultured up to passage 5 were utilized for all subsequent experiments.

### Toxicity assay

Viability of HUVECs and HUVSMCs cultured with increasing GA concentrations for 6 and 24 h was assessed with a PresoBlue cell Viability Reagent (Invitrogen, Frederick, U.S.) according to the manufacturer's instructions. Fluorescence readings were taken at 544 nm/590 nm excitation and emission wavelength respectively which correspond directly to the amount of viable cells.

### Biomechanical stretch stimulation of cells in culture

To expose HUVECs to biomechanical stretch, cells were cultured on BioFlex Collagen type I 6-well plates (Flexcell, Hillsborough, NC, USA) pre-coated with Geltrex® (basement membrane surrogate, 1:10 in cell media) for 1 h at 37°C. One day prior to stretch, the endothelial supplement content of the media was reduced to half and diluted in the M199 media supplemented with 12.5% FCS, 25 U/ml penicillin, 25 μg/ml streptomycin and 0.125 μg/ml Fungizone® antimycotic. Cell monolayers were treated with GA dissolved in DMSO at 20 μM or 40 μM final concentration or equivalent volume of DMSO vehicle control. Cyclic stretch was applied 1.5 h later using a microprocessor controlled vacuum pump (FX-3000 FlexerCell Strain Unit, Flexcell, Hillsborough, NC) with 15% cyclic elastomer elongation at frequency of 0.5 Hz. Cyclic, as opposed to static, elongation is needed to prevent the cells from evading the biomechanical stimulus through rearranging their focal contacts. To expose HUVSMCs to biomechanical stretch, cells were cultured on BioFlex Collagen type I 6-well plates (Flexcell, NC, USA) in 15% FCS, DMEM media. One day prior to stretch the cell media was exchanged to pure DMEM media in order to stabilize HUVSMCs phenotype in the absence of serum. Cell monolayers were treated with GA (1 h at 5 μM final concentration) or an equivalent volume of DMSO vehicle control. Cyclic stretch was applied using a microprocessor controlled vacuum pump (FX-5000 FlexerCell Strain Unit, Flexcell, NC) with 15% cyclic elastomer elongation at frequency of 0.5 Hz.

### Immunofluorescence-based detection of gelatinase activity

HUVECs and HUVSMCs were fixed with methanol for 15 min at 4°C, air-dried and blocked with Casein/BSA block buffer (0.25% Casein, 0.1% BSA, 50 mM Tris, pH 7.6) for 30 min. Determination of gelatinase activity was performed by incubating methanol-fixed cells with fluorescein-conjugated DQ-gelatin (EnzChek gelatinase assay kit; Molecular Probes/Invitrogen, Leiden, Netherlands) for 1 h at 37°C as per manufacturer's instructions. Nuclei were visualized by counterstaining with DAPI (2 μg/mL, diluted in PBS) for 10 min and the cells were then washed and mounted with Mowiol 4-88 (Fluka). Fluorescence intensity was recorded using a fluorescence microscope IX83 (Olympus) and quantified by using the Cell^∧^R software (Olympus, Hamburg, Germany) analyzing at 4–5 different regions of the specimen per experimental group. Exposure times during digital imaging were kept constant.

### DNA microarray analysis

HUVECs from three different donors were stimulated with GA (40 μM) or DMSO as solvent control and exposed to biomechanical stretch for 6 h. RNA was isolated and processed for DNA microarray analysis according to manufacturers' instructions: Gene expression profiling was performed using the GeneChip® Human Genome Array from Affymetrix. After RNA isolation RNA was purified using the RNA Clean-Up and Concentration Micro Kit. cDNA synthesis was performed using the SuperScript Choice System according to the recommendations of the manufacturer. Using ENZO BioArray HighYield RNA Transcript Labeling Kit biotin-labeled cRNA was produced. Standard protocol from Affymetrix was used for the *in vitro* transcription (IVT). Quantification of cRNA was performed by spectrophotometric analysis with an A260/A280 ratio of 1.9–2.1. Fragmentation of the cRNA was achieved using Affymetrix defined protocol. For gene expression profiling, labeled and fragmented cRNA was hybridized to Affymetrix Hugene-2_0-st microarrays with an incubation of 16 h at 45°C. The Affymetrix fluidics station 450 was used to wash the microarrays, scanning was performed with Affymetrix Genechip scanner 3000. A custom CDF version 20 with Entrez-based gene definitions was used to annotate the arrays. Raw fluorescence intensity values were normalized applying quantile normalization. Differential gene expression analysis was performed with one-way analysis of variance (ANOVA) using the software package JMP10 Genomics version 6 from SAS (SAS Institute). A false positive rate of *a* = 0.05 with FDR correction was taken as the level of significance.

### Collagen-gel based migration/invasion assays

HUVEC spheroids of defined cell number were generated as described previously (Heiss et al., 2015). In brief, HUVECs were suspended in the culture medium containing 0.25% (w/v) methylcellulose and seeded into non-adherent round bottom 96-well plates overnight allowing for the formation of a single, well-defined round spheroid in each of the tissue culture wells (500 cells per spheroid were seeded). Spheroids were collected the next day and embedded into liquid collagen gels which were allowed to polymerize. GA or DMSO control were dissolved in EC basal media (Promocell, Heidelberg, Germany) containing VEGF (25 ng/mL) and pipetted onto the gels (100 μl total volume). The gels were incubated at 37°C, 5% CO2 for 24 h and the angiogenesis was quantitated by measuring the length and the number of the sprouts (calculated as cumulative sprout length) that had grown out of each spheroid obtained by microscopy imaging using Olympus CellD software (Shinjuku, Tokyo, Japan).

HUVSMCs were suspended in growth medium containing 0.25% (w/v) methylcellulose and seeded onto non-adherent round bottom 96-well plates overnight allowing for the formation of a single, well-defined a round spheroids which were suspended in collagen gels as described earlier (Pfisterer et al., 2014b). GA or DMSO control were dissolved in DMEM cell media and pipetted on top of the gels (100 μl total volume). The gels were incubated at 37°C, 5% CO2 for 24 h and the invasive properties of SMCs were quantitated by measuring the length and number of SMCs projections (calculated as cumulative length) that had grown out of each spheroid obtained by bright field microscopy imaging and quantification using Olympus CellD software (Shinjuku, Tokyo, Japan).

### Proliferation cell count

HUVECs/HUVSMCs were treated with various concentrations of GA or equivalent volume of the DMSO vehicle control at ~30% confluency and allowed to grow for an additional 24 h. Light microscopy images from the same areas of the well were obtained immediately after pre-treatment as well as 24 h and the total number of cells in each field of view was quantified with the help of ImageJ software (NIH, Bethesda, MD, USA). The cell number was converted into doubling time using the following formula:

$$\begin{array}{l} \text{Doubing time}=\;\frac{\text{time in culture}*\text{Log }\left(2\right)}{\text{Log }\left(\text{final cell \# }\right)-\text{Log }\left(\text{initial cell \#}\right)\;} \end{array}$$

TissueGnostics/TissueQuest microscopy and software were utilized to simultaneously automatically assess the number of Ki67-positive cells. In brief, HUVECs were fixed with methanol for 15 min at 4°C, air-dried and blocked with Casein/BSA block buffer for 30 min, incubated with the anti-Ki67 antibody overnight, washed with PBS and nuclei were visualized by DAPI staining, automatically detected and defined as region of interest (ROI). ROI-associated ki67 fluorescence was automatically detected and expressed as percentage fluorescence-positive nuclei with fluorescence signal above background using TissueFAXS microscopy (TissueGnostics AG, Austria) and TissueQuest (TissueGnostics AG, Austria) software.

### AP-1 binding assessment

HUVECs were pre-treated with GA (40 μM) or DMSO vehicle control subject to 6 h of biomechanical stretch as described in the previous section. Preparation of nuclear extracts from the cultured cells and subsequent non-denaturing 4% polyacrylamide gel electrophoresis was carried out as described previously (Krzesz et al., 1999). The double-stranded gel shift oligonucleotides (ODN; cat. no: sc-2501 AC; Santa Cruz Biotechnology, Heidelberg, Germany) for AP-1 consensus sequence (5′-CGCTTGATGACTCAGCCGGAA-3′) were end-labeled with [γ-32P]ATP by using the 5′-end labeling kit from Amersham Pharmacia Biotech. Typically the binding mixture contained 5 μg of nuclear extract, 20,000 cpm of the 32P-labeled oligonucleotide probe (0.5 ng), 1 μg poly[d(I-C)] and 1.33 mM DL-dithiotreitol in a total volume of 15 μl binding buffer. The resulting protein-DNA complexes were analyzed by non-denaturing polyacrylamide gel (4%) electrophoresis and autoradiography by exposing the dried gels to Kodak X-OMAT AR X-ray film (Sigma-Aldrich).

### Assessment of the MMP2/9 activity

The impact of GA on the activity of gelatinases was determined by measuring the MMP2 or MMP9-mediated turnover of the gelatinase-specific substrate BML-P125-9090. In brief, GA or an equivalent volume of solvent (DMSO) were mixed with recombinant human MMP2 or MMP9 (90 mU/μl) and incubated for 30 min at 37°C. After adding the substrate (2 mM), absorption of the produced chromogen was acquired at A_412_ every minute according to manufacturer's instructions (MMP2/9 colorimetric drug discovery kits, Enzo Life Sciences, USA).

### Animal model

All animal studies were approved by the Regional Council Karlsruhe and carried out in accordance with the Guide for the Care and Use of Laboratory Animals published by the US National Institutes of Health (NIH Publication No. 85-23, revised 1996). Ligation of mouse auricle veins was performed as described earlier (North and Sanders, 1958; Feldner et al., 2011; Pfisterer et al., 2014b). In brief, NMRI male mice (Janvier; at least 12 weeks of age) were anesthetized with isoflurane and one of the three first order veins was ligated using a surgical thread (silk, 7.0, Ethicon, Norderstedt). Due to the highly collateralized auricle vasculature, this procedure does not lead to ischemia or necrosis. Remodeling of collateral veins was documented on a daily basis by using a high-resolution digital camera (Olympus, Hamburg, Germany) and the PSI-System (Perimed, Stockholm) to survey changes in local perfusion. Images were morphometrically analyzed using the software Image J (NIH, Bethesda, MD, USA). A cream formulation (Unguentum emulsificans aquosum) containing GA (5 μg/ear, roughly matches an effective concentration of 20–40 μM GA considering tissue/cartilage volume, penetration time and drainage) or DMSO vehicle control (1% by weight) was transdermally administered to the auricles of mice 1 day prior as well as every second day after ligation surgery Four days upon ligation, mice were sacrificed and perfused with Ringer solution and zinc-fixative. Mouse auricles were dissected and processed for paraffin embedding and histological examination.

### Perfusion of isolated mouse veins

Animals were sacrificed and the facial and saphenous veins (~1 cm long segments, diameter: 50–100 μm) were extracted and inserted into a perfusion chamber (Culture Myograph, DMT, Copenhagen, Denmark). The chambers were placed in an incubator at 37°C and 5% CO_2_, and the free end of blood vessel segments were tied off with a suture. This allowed for an increase in intraluminal hydrostatic pressure of the vessels upon continuous stretch distension at a transmural pressure difference of 4 or 16 mmHg without the application of flow (varicosis-inducing conditions: ΔP: 16 mmHg; physiological (control) condition: ΔP: 4 mmHg). The vessels were continuously stretch-distended for 18 h in Panserin 401 media (PAN-Biotech, Aidenbach, Germany) supplemented with 50 U/ml penicillin, 50 μg/ml streptomycin and 0.25 μg/ml Fungizone® antimycotic in the presence of GA (10 μM) or the equivalent volume of DMSO vehicle control. After perfusion, vessel segments were fixed in Dent's fixative (4°C for 24 h; 80% methanol, 20% DMSO) and subsequently processed for whole-mount immunofluorescence analyses.

### Whole-mount immunofluorescence

*Ex vivo* stretch distended vein segments fixed in Dent's fixative were rehydrated in PBS with decreasing methanol content (75, 50, 25%) for 10 min, washed and incubated with a fluorescein-conjugated DQ-gelatin for 1 h at 37°C, washed and counterstained with DAPI for 30 min for nuclear visualization. Vessels were washed again with PBS for 30 min and mounted longitudinally onto glass slides in the presence of Mowiol 4-88 mounting media. Fluorescence signal intensity was captured and quantified using the Cell^∧^R software (Olympus, Hamburg, Germany).

### PSI imaging

Blood perfusion of an area of ~20 × 20 mm containing a spider vein (left leg, knee level) was made from one healthy female volunteer using the PeriCam PSI blood perfusion imager placed at a 10 cm distance perpendicular to the skin (image resolution: 20 μm/pixel; Camera resolution: 752 × 580 pixels). This system is based on the Laser Speckle Contrast Analysis (LASCA) technology which allows for visualization of blood perfusion (Perfusion Units, PU) of the superficial organs such as skin. Informed (written) consent in accordance with the Declaration of Helsinki has been obtained from the volunteer for imaging of a spider vein as well as online open-access publication of the information/images.

### Statistical analysis

As normality tests of small samples (<10) have little power to discriminate between Gaussian and non-gaussian populations, we made assumptions about their distribution in a given basic population by considering the variability/scatter/source of scatter of data from previous experiments assessing the same variables(Feldner et al., 2011; Eschrich et al., 2016; Kuk et al., 2017). For the presented experimental data, we expect a Gaussian distribution. Consequently, differences between 2 matched experimental groups were analyzed by unpaired Student's *t*-test, with a probability value of *p* < 0.05 considered statistically significant. Differences of one parameter among 3 or more experimental groups were analyzed by one-way ANOVA, followed by a Tukey-Kramer multiple comparisons test, with a probability value of *p* < 0.05 considered statistically significant. If not stated otherwise, bars represent the mean ± SD of an independent experiments based on cells/veins from individual donors/mice.

## Results

### Increase in the venous pressure is sufficient to drive the remodeling of superficial veins

**Figure 1:**
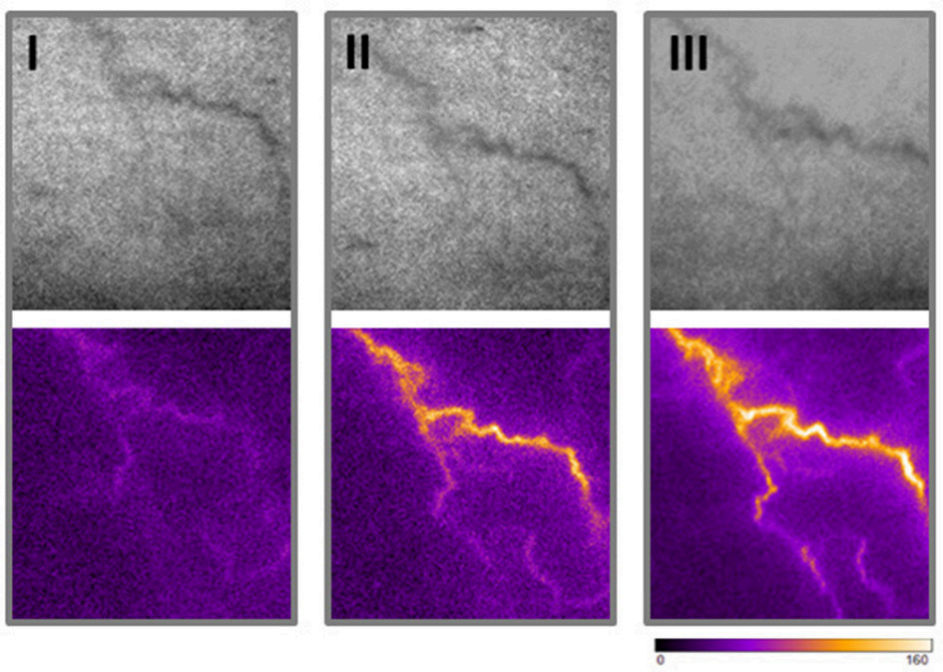
Morphometrical and functional analyses of superficial veins. Blood perfusion of one spider vein (left leg, knee level) was made from a healthy female volunteer using the PeriCam PSI blood perfusion imager (Superficial image: top panel; Perfusion measurement: bottom panel). An increase in blood perfusion of a spider vein was observed when the volunteer actively contract muscles of the leg (II) as opposed to the low perfusion level while standing still (I). Morphology and perfusion (upon muscle contraction) of the same spider vein was assessed 19.5 months later in a follow up recording (III).

Superficial veins or venules are connected to the deeper venous plexus (Somjen, 1995; Meissner, 2005) and may thus be exposed to enhanced biomechanical load if the venous filling pressure increases. As a consequence, they may remodel to form bulged, tortuous and dilated spider veins in the superficial skin. We exemplarily highlighted the direct communication of superficial spider veins and the deeper venous plexus by measuring their perfusion during a muscle contraction-induced increase in venous blood pressure. An increase in spider vein perfusion was observed when the volunteer contract the muscles of the leg as opposed to the low perfusion levels after prolonged standing without any muscle contraction (Figure 1). Further, follow up imaging analysis of a spider vein from the human volunteer (no changes in weight or health status) did not exhibit any signs of morphological change (growth or progression) 19.5 months later (Figure 1) of the observed spider vein.

### The impact of glycyrrhetinic acid on properties of cultured venous vessel wall cells

**Figure 2:**
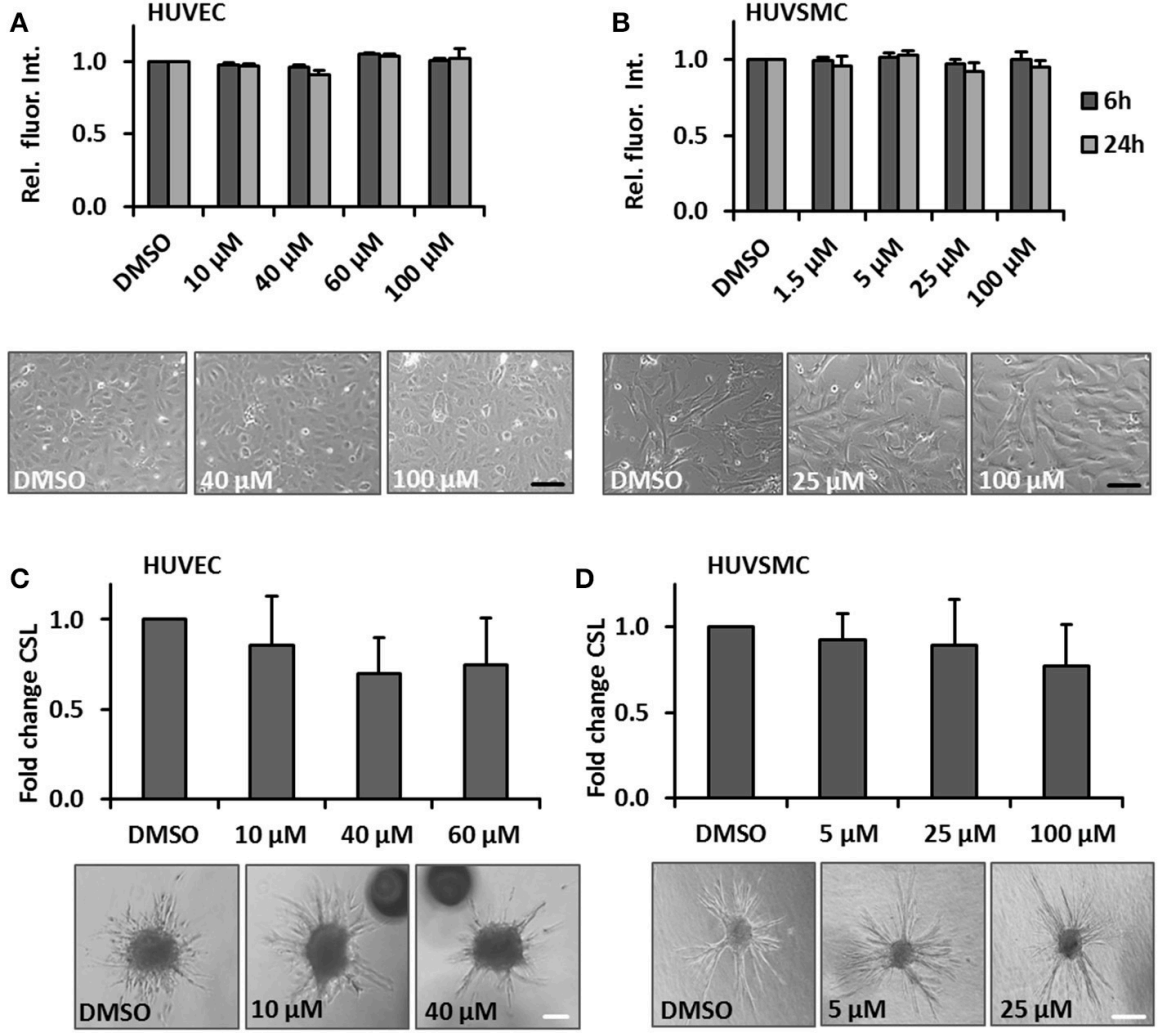
GA does not affect viability and migration of cultured HUVECs or HUVSMCs. HUVECs **(A)** or HUVSMCs **(B)** were treated with the indicated concentrations of GA or DMSO vehicle control for 6 and 24 h. Cell viability was determined by the PrestoBlue fluorescence-based assay. The fluorescence intensity readout for each treatment group was normalized to the DMSO vehicle treated control (set to 1). Representative phase contrast microscopy images are shown (*p* > 0.05 for all comparisons, *n* = 3–4; scale bars: 100 μm). HUVEC **(C)** or HUVSMCs **(D)** spheroids were embedded into a collagen matrix and treated with indicated concentrations of GA or DMSO vehicle control (HUVEC sprouting was induced by stimulation with 25 ng/ml VEGF-A). The spheroids were visualized and imaged 24 h later. The cumulative sprout length originating from individual spheroids treated with GA was quantified as a relative fold change compared to the DMSO treated controls (set to 1), (*p* > 0.05 for all comparisons, *n* = 3–4; scale bars: 50 μm).

**Figure 3:**
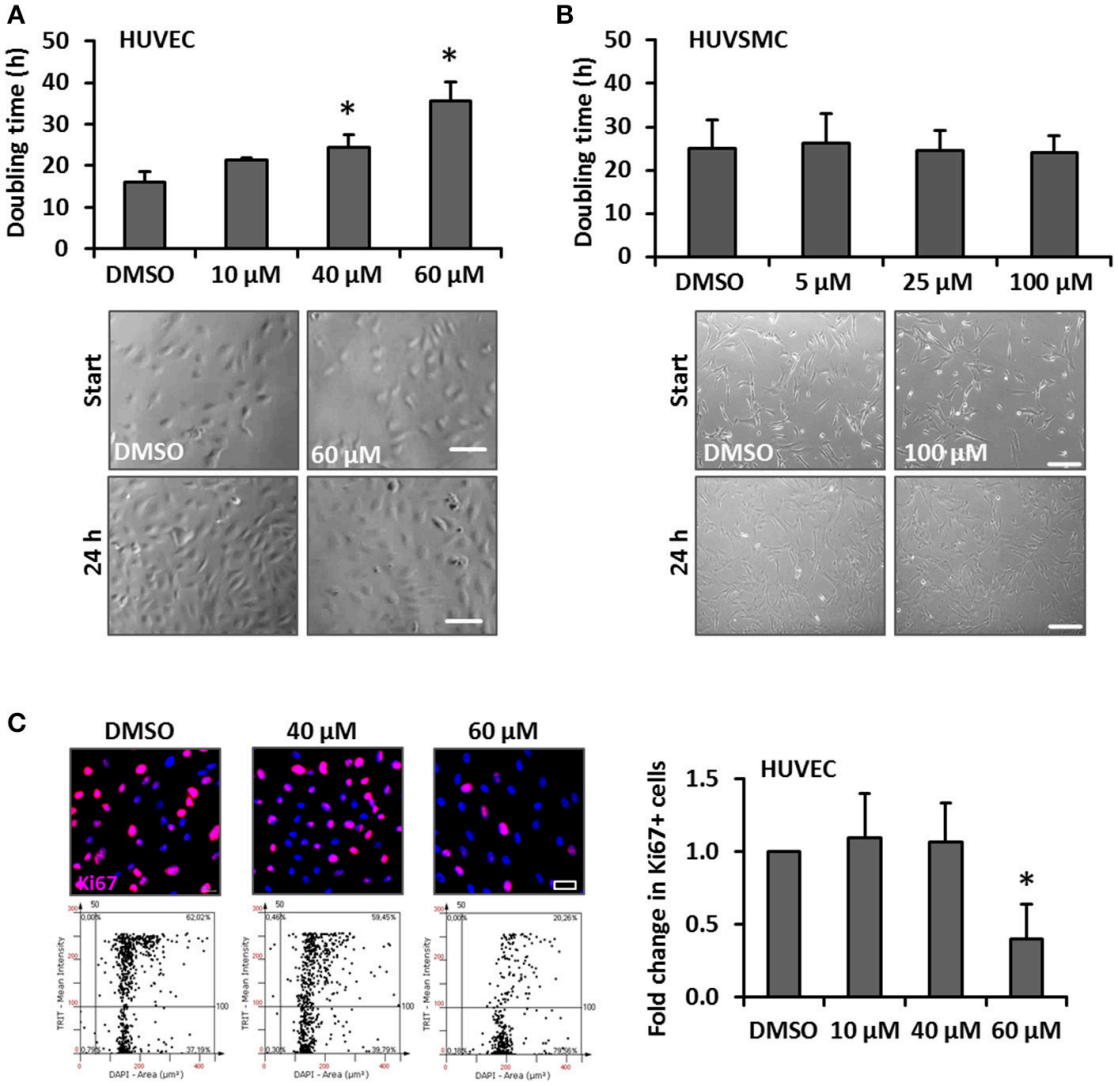
Impact of GA on HUVEC and HUVSMC proliferation. Proliferation of HUVECs **(A)** and HUVSMCs **(B)** was carried out by counting the number of cells within 5 defined MFV per experimental group and calculated as time (h) needed to double the cell population. Cells were treated with the indicated concentrations of GA or an equivalent volume of DMSO vehicle control (**A**, *p < 0.05 vs. DMSO, *n* = 3; **B**, *p* > 0.05 for all comparisons, *n* = 4). Representative phase contrast images showing the original cell density (start) and after 24 h (**A**, scale bar: 100 μm; **B**, scale bar: 200 μm). Fold change of Ki67-positive (Ki67+) HUVECs treated with DMSO or the indicated concentrations of GA was obtained using the automated immunofluorescence analysis (representative images are shown in **C**, scale bar: 20 μm), demonstrated in the corresponding scattergrams (**C**, lower panel of representative images) and calculated as fold change of Ki67+ HUVECs [*p < 0.05 vs. DMSO (set to 1), *n* = 4].

One hallmark of spider veins is their increased diameter and given that blood vessel growth almost always relies on the proliferation of ECs and SMCs, we hypothesized that cream formulations containing bioactive compounds interfering with biomechanically evoked activation of vascular cells may attenuate the development of spider veins. GA was selected due to its frequent usage in personal care products and anti-proliferative features (Huang et al., 2014; Tang et al., 2015; Zhu et al., 2015). In order to assess possible cytotoxic effects of this compound, the viability of human venous ECs and SMCs exposed to increasing concentrations of GA was analyzed. The selected GA concentrations were based on reports from the literature (e.g., Matchkov et al., 2004; Behringer et al., 2012; Kizub et al., 2016) and did not affect the cellular viability of HUVECs and HUVSMCs in culture (Figures 2A,B respectively). In addition, we tested whether GA may interfere with the cellular motility required for angiogenesis (e.g., in the context of wound healing processes) and structural maintenance of blood vessels. Sprouting assays revealed that GA did not interfere with the migratory capacity of ECs and SMCs (Figures 2C,D). Finally, we investigated whether GA affects the proliferation of these cells by determining their doubling time upon GA treatment. While HUVEC growth (Figure 3A) was inhibited under these conditions, HUVSMCs proliferation was not affected (Figure 3B). The former result was partially confirmed by automated immunofluorescence-based detection of the proliferation marker Ki67 in ECs (Figure 3C).

### Glycyrrhetinic acid attenuates AP-1 activity in biomechanically stimulated venous endothelial cells

**Figure 4:**
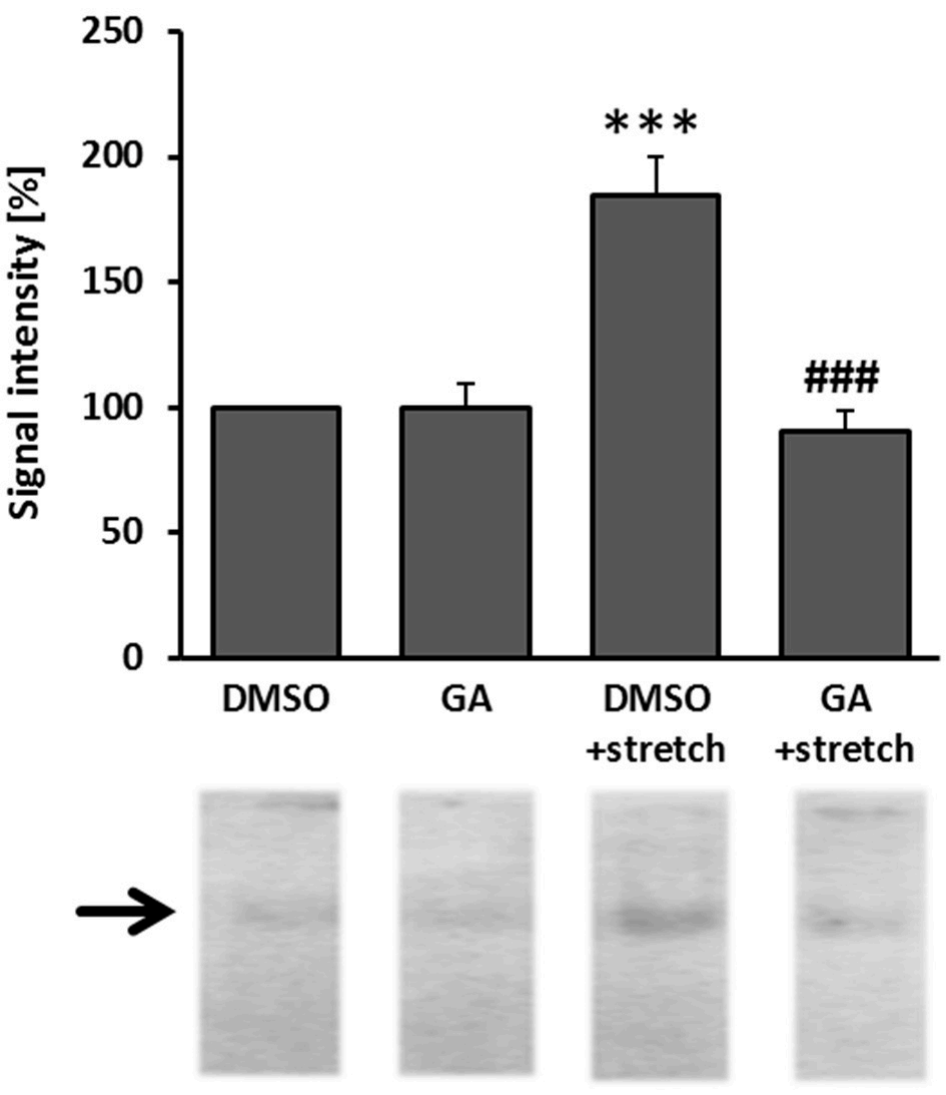
GA attenuated the capacity of AP-1 binding to DNA in biomechanically stimulated HUVECs. HUVECs were treated with GA (40 μM) or DMSO control vehicle for 1.5 h and subjected to biomechanical stretch (15% cyclic elongation at 0.5 Hz) for 6 h. Binding of the mechanosensitive transcription factor AP-1 was assessed in an EMSA assay (***p < 0.001 vs. DMSO, ^###^*p* < 0.001 vs. DMSO stretch, *n* = 3). Signal intensity (gray value) of DMSO-treated cells was set to 100%.

As has been repeatedly shown by results of our group, biomechanical wall stress or stretch of venous endothelial and SMCs is sufficient to promote their activation and acts as a major determinant of venous remodeling (Feldner et al., 2011; Pfisterer et al., 2014a). Considering this and the putative sensitivity of ECs to GA treatment (Figure 3), we next investigated whether GA may also attenuate prototypic responses to biomechanical stress such as activation of the mechanoresponsive transcription factor AP-1. Its activity was analyzed by determining the binding of radioactively-labeled DNA fragments mimicking the AP-1 binding site to electrophoretically separated proteins of endothelial (nuclear) protein lysates. The corresponding results indicated an elevated AP-1 binding capacity in the nuclei of biomechanically stressed ECs which was fully absent upon GA treatment (Figure 4 and Figure S1). However, consequent detailed transcriptome analyses of stretch-exposed ECs (access# for full microarray data via Gene Expression Omnibus (GEO): GSE110335) indicated only minor effects of GA on AP-1 target gene expression (Table 1). Furthermore, signaling events mediated through MAP kinases in biomechanically activated HUVECs were not inhibited by GA (Figure S2).

**Table 1:** Assessment of changes in the transcriptome of GA-treated HUVECs exposed to biomechanical stretch.

**GeneSymbol**	**GeneName**	**Ensembl ID**	**Fold change (log2)**	**Adj. *p*-value**
ZBTB43	Zinc finger and BTB domain containing 43	ENSG00000169155	0.446	0.020
PHLPP2	PH domain and leucine rich repeat protein	ENSG00000040199	0.424	0.043
TMOD2	Tropomodulin 2 (neuronal)	ENSG00000128872	0.391	0.012
ALG1	ALG1, chitobiosyldiphosphodolichol beta-	ENSG00000033011	0.383	0.004
SNX18	Sorting nexin 18	ENSG00000178996	0.339	0.003
CTSF	Cathepsin F	ENSG00000174080	0.338	0.025
ATG10	Autophagy related 10	ENSG00000152348	0.313	0.004
LACTBL1	Lactamase, beta-like 1	ENSG00000215906	−0.338	0.043
DEFB113	Defensin, beta 113	ENSG00000214642	−0.368	0.018
SNORA11B	Small nucleolar RNA, H/ACA box 11B	ENSG00000221102	−0.379	0.039
IQCD	IQ motif containing D	ENSG00000166578	−0.419	0.019
RNF126	Ring finger protein 126	ENSG00000070423	−0.687	0.021
DDX11L9	DEAD/H (Asp-Glu-Ala-Asp/His) box helicas	ENSG00000248472	−0.736	0.050

*HUVECs from three different donors were treated with GA (40 μM) or an equivalent volume of DMSO vehicle control for 1.5 h and subjected to biomechanical stretch (6 h). mRNA was isolated and processed for microarray-based analysis. Genes which were affected by GA treatment (vs. DMSO) were selected based on the following criteria: fold change (log2) > 0.3, p < 0.05 for adjusted p-value (ANOVA), n = 3. Only significantly regulated transcripts were listed (green: elevated transcript level, red: decreased transcript level). An altered gene expression pattern was not detected as evidenced by gene set enrichment analyses (GSEA)*.

### Gelatinase activity of mechanostimulated venous cells is inhibited by glycyrrhetinic acid

**Figure 5:**
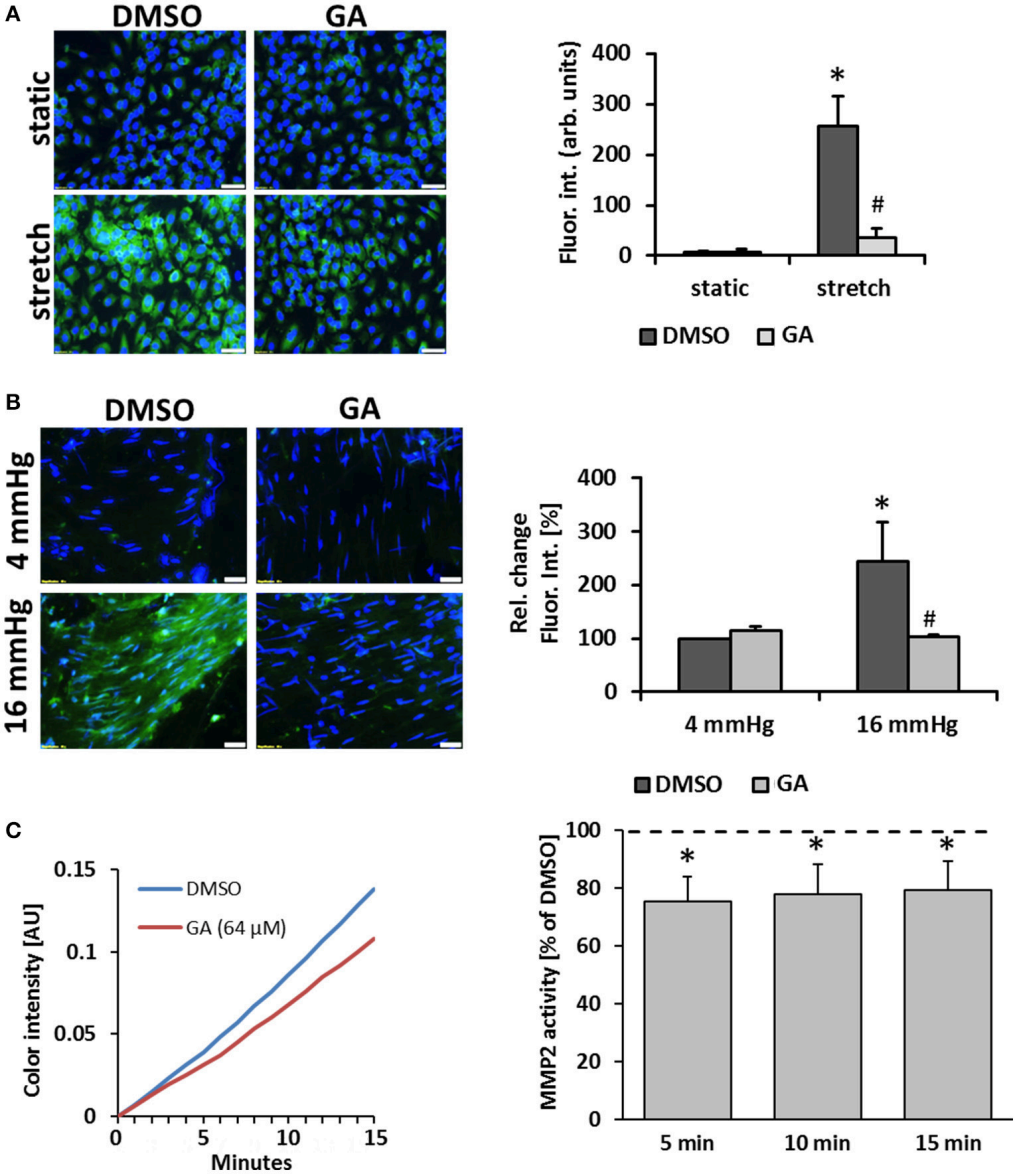
GA attenuates gelatinase activity in biomechanically stimulated HUVECs or mouse veins. HUVECs were exposed to biomechanical stretch (15% cyclic elongation at 0.5 Hz) for 24 h with or without GA pretreatment (1.5 h, 20 μM) followed by quantitative analysis of gelatinase activity (**A**, green fluorescence, *p < 0.05 static DMSO vs. stretch DMSO; ^#^*p* < 0.05 stretch DMSO vs. stretch GA, bars represent the mean ± SD of 4 MFV of 1 representative out of 4 experiments with comparable results; scale bars: 50 μm). Mouse vein segments were pretreated with GA or an equivalent volume of DMSO for 1.5 h and exposed to basal (4 mmHg) and increased (16 mmHg) intraluminal pressures for 18 h. Gelatinase activity (green fluorescence) was assessed in a whole-mount staining procedure; nuclei were counterstained with DAPI [**B**, *p < 0.05 vs. DMSO (4 mmHg); ^#^*p* < 0.05 vs. DMSO (16 mmHg), *n* = 5; scale bars: 50 μm]. The capacity of GA to interfere with the activity of recombinant human MMP2 was assessed by applying a colorimetric assay **(C)**. GA (64 μM) lowered the MMP2 activity as compared to the corresponding DMSO solvent control (**C**, representative curves showing the chromogen production over time; bars represent the mean percent of chromogen production as compared to solvent control ± SD at 3 time points [*p < 0.05 vs. solvent control (set to 100%, dotted line), *n* = 4]).

Another prototypic response of ECs subject to biomechanical stress is the activation of proteases which promote degradation of extracellular matrix components as a prerequisite for further remodeling of the vessel wall (Kowalewski et al., 2004; Raffetto and Khalil, 2008). In this context, we assumed that GA may inhibit the stretch-mediated expression of the metalloproteinases MMP2 and MMP9 in ECs but this was neither evidenced by the microarray data nor additional immunofluorescence studies detecting MMP2/9 protein in stretch-stimulated ECs upon GA treatment (Figure S3). However, we revealed that gelatinase activity (the combined activity of “gelatinases” such as MMP2 and MMP9) in stretch-exposed ECs is blunted upon treatment with GA (Figure 5A). Similar results were obtained with HUVSMCs (Figure S4). To monitor this GA-mediated effect under physiological conditions, mouse vein segments were exposed to physiological (4 mmHg) and supraphysiological (16 mmHg) intraluminal hydrostatic pressure that forces distension of the venous vessel wall. In line with the former results, treatment with GA inhibited the pressure-induced increase in the gelatinase activity (Figure 5B). As the expression and protein level of gelatinases was not affected by GA in biomechanically stressed HUVEC, we hypothesized that GA may directly interfere with their enzymatic activity. This assumption was scrutinized by exposing recombinant human MMP2 or MMP9 to GA to assess the turnover of a gelatinase-specific substrate. Initial screenings revealed slight inhibitory effects of GA for MMP2 and MMP9 activity at concentrations of 64 and 32 μM, respectively (Figure S5). While the effect on MMP9 turned out to be not significant (data not shown), additional analyses confirmed a putative impairment of MMP2 activity by GA (Figure 5C).

### Transdermal application of glycyrrhetinic acid prevents venous remodeling in the mouse auricle

**Figure 6:**
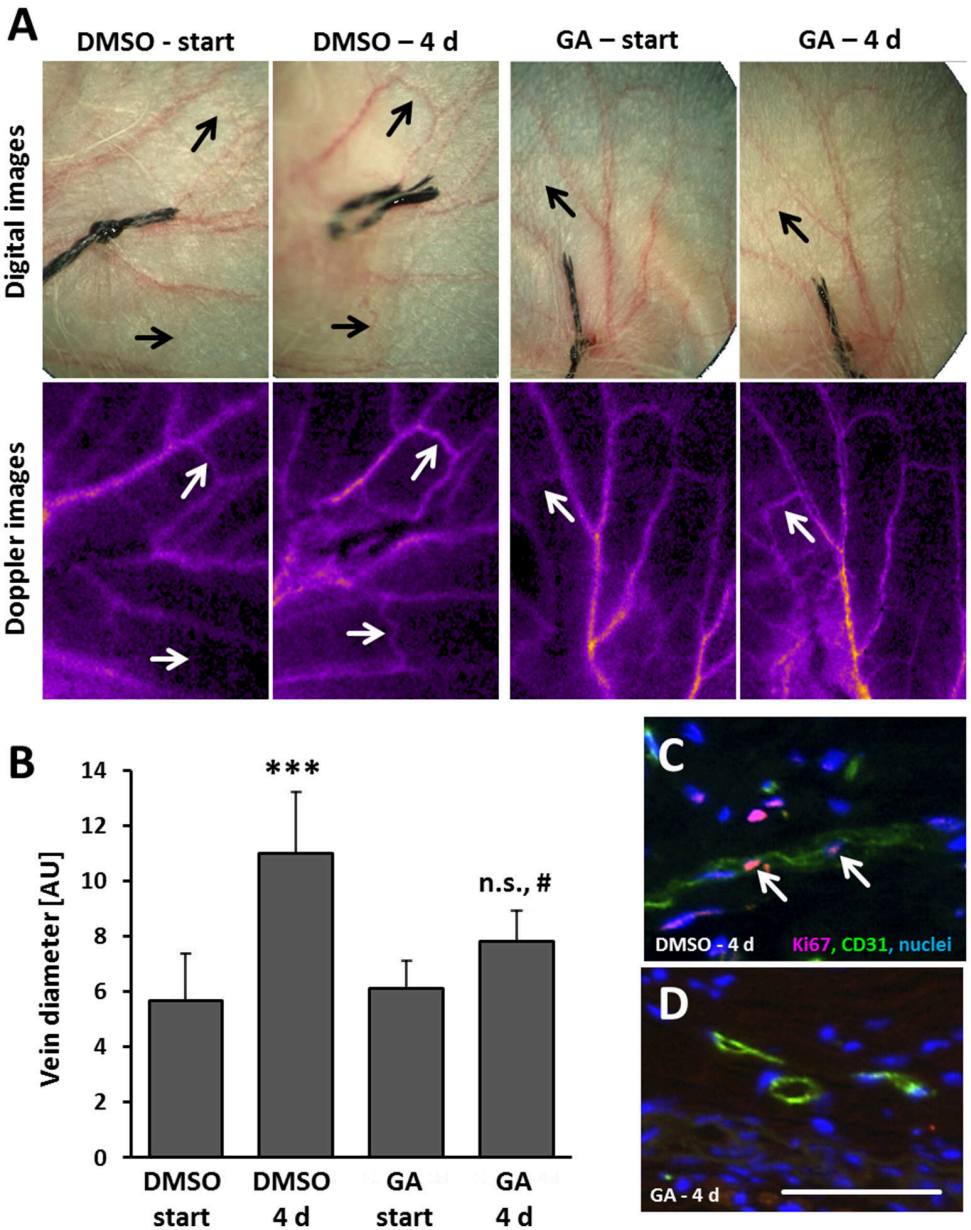
GA attenuates formation of spider veins in the mouse auricle. Mouse auricles were treated with GA (~5 μg/ear) or DMSO (corresponding vehicle volume) applied as cream formulations before ligation and on every other day. Ligation of a central auricle vein was utilized to locally increase the venous volume load. Morphological and functional changes in the venous system was recorded by digital (**A**, upper panel) imaging of the auricles right after (start) and 4 days after ligation. Adequate occlusion and alteration of the flow profile was controlled by laser-Doppler imaging (**A**, lower panel). Diameter of remodeling veins (**A**, arrows) far distant from the ligation site was assessed immediately after (start) and 4 days (4 d) after ligation in living animals [**B**, ***p < 0.001 DMSO start vs. DMSO 4 d; ^#^*p* < 0.05 GA 4 d vs. DMSO 4 d, not significant (n.s.) GA 4 d vs. GA start, *n* = 6-7, fully dilated veins were compared only]. Exemplary images of the IF detection of Ki67 in a DMSO- (**C**, arrows) and GA-treated **(D)** remodeling veins along with an endothelial cell marker CD31 (scale bar: 50 μm).

As evidenced by our findings, GA may diminish remodeling of superficial veins at least by interfering with (i) EC proliferation, (ii) AP-1 binding capacity, and (iii) the gelatinase activity. To scrutinize the relevance of these findings in the context of venous remodeling *in vivo*, a mouse model was applied that triggers remodeling of superficial veins by locally increasing venous pressure (Feldner et al., 2011). By ligating a single central vein of the auricle, the connected venous network is immediately inflated indicating a sudden increase in pressure. Previous perfusion analyses of affected veins revealed a marginally elevated perfusion at that time (Pfisterer et al., 2014b). Originating from the dilated state, remodeling of these veins almost doubles their diameter (Figures 6A,B). Transdermal application of GA on every other day significantly diminished the enlargement of auricle veins (Figures 6A,B) and was accompanied by a diminished EC proliferation (Figures 6C,D).

## Discussion

Veins are constantly exposed to biomechanical forces exerted by the movement of blood inside the vessel and depending on the magnitude and duration this may stabilize or “activate” the cells comprising the venous vessel wall (Pfisterer et al., 2014a). The initiating triggers for venous remodeling processes associated with spider-vein formation in particular have been a subject of debate. In this context, we assume that the increased filling pressure acts as the initial trigger for venous ECs and SMCs to respond in a maladaptive fashion. Indeed, risk factors causing increased pressure in the abdomen (e.g., obesity, pregnancy) may initiate and promote venous remodeling (Sisto et al., 1995; Pfisterer et al., 2014a). In line with this idea, an increase in venous filling pressure alone supports enlargement and dilatation of superficial veins in mice (North and Sanders, 1958; Feldner et al., 2011; Kuk et al., 2017). In humans, pressure/wall stress-induced remodeling of superficial veins may in fact be explained by direct connection to the deep venous plexus as was evidenced by the increase in perfusion of a spider vein upon muscle contraction-mediated pressure increase in the deep veins. From a therapeutic point of view, the interference with wall stress-mediated mechanisms driving venous remodeling in the skin may be achieved by transdermal application of bioactive compounds targeting rate limiting steps of this process. In this study, we selected GA as a suitable compound due to its usability in skin care products and its properties counteracting excessive cellular activation.

While not much is known of the effects of GA on ECs and SMCs in the context of their stretch-dependent activation, GA has been shown to non-specifically block gap junctions, e.g., composed of connexin-43 in vascular SMCs (Matchkov et al., 2004; Sobieszczyk et al., 2010). However, under the chosen experimental conditions, GA had only little impact on the SMC phenotype. Effects of GA on vascular ECs were also examined previously particularly in the context of pro-inflammatory monocyte adhesion (Chang et al., 2010) upon stimulation with TNF-α. Similar to the current findings, treatment of ECs was found to be well tolerated under basal conditions. However, according to its anti-proliferative impact on tumor cells (Huang et al., 2014; Tang et al., 2015; Zhu et al., 2015), higher concentrations of GA exhibited reduced proliferation of cultured ECs but not SMCs—albeit the origin of this effect remaining unknown.

Surprisingly, GA appears to robustly interfere with the function of AP-1 in stretch-exposed ECs which, however, did not much affect their transcriptional profile. Other reports suggest that in TNF-α-stimulated ECs GA may also influence the activity of NFκB (Chang et al., 2010)—another transcription factor that is activated in biomechanically stimulated ECs (Du et al., 1995; Zhao et al., 2009). NF-κB was found to be attenuated upon GA treatment through blockade of IκB degradation and hence nuclear translocation of NF-κB p65 as well as by reducing the DNA-binding activity of NF-κB (Chang et al., 2010). Increases in phospho(activated)-JNK levels (pro-mitogenic mitogen activated kinase) reported upon TNF-α induction were also attenuated in HUVECs subject to GA (up to 50 μM) treatment without affecting TNF-α-induced phospho-p38 or phospho-ERK levels (Chang et al., 2010). While GA may thus in principle affect the activity of transcription factors and this may in fact influence the outcome of venous remodeling processes in the long run, based on the current finding this effect had only a minor immediate impact on the transcriptional profile or gene expression pattern of cultured ECs exposed to biomechanical stress.

Despite the ambivalent effects of GA observed so far, it robustly inhibited the capacity of both human and mouse ECs and SMCs to degrade gelatin-based matrices. This inhibitory feature may in fact attenuate the renovation of the extracellular matrix in the context of venous remodeling which is usually associated with increased activity of matrix metalloproteinases (MMP-2/9) in animal models (Pascarella et al., 2005; Raffetto et al., 2008; Eschrich et al., 2016; Kuk et al., 2017) and diseased human veins (Woodside et al., 2003; Kowalewski et al., 2004; Nomura et al., 2005). As our findings did not reveal a GA-mediated down-regulation of the MMP2/9 expression, its observed gelatinase-inhibiting effect may be based on its direct interference with the enzymatic activity as has been described for many other enzymes such as 11beta-hydroxysteroid dehydrogenase (Monder et al., 1989), tyrosinase (Um et al., 2003), hyaluronidase (Hertel et al., 2006). In fact, our data indicated a weak inhibitory impact of GA on the enzymatic activity of MMP2 which may in principle explain for a decreased cellular MMP2 activity. While *in vitro* only high concentrations were able to inhibit the activity of a gelatinase, prolonged exposure of cells to GA and further inhibitory effects on enzymes crucial for activating MMPs such as MT1-MMP (Sato et al., 1994) may amplify this effect and increase the efficacy of GA *in vivo*.

Although the mechanistic details of the GA-induced inhibitory effects remain unknown, collectively they may interfere with the capacity of biomechanically stressed ECs and partly SMCs to adequately promote the structural reorganization of a superficial vein. In fact, this study shows for the first time that GA attenuates venous remodeling *in vivo* when applied as a topical cream formulation at 5 μg per auricle/day. This dose is below the limit established in previous studies utilizing transdermal application of GA in mice either in the form of an intradermal injection or topical administration (Inoue et al., 1989; Wang et al., 1991; Akasaka et al., 2011; Kong et al., 2015).

Pharmacokinetic and pharmacodynamic properties of GA have been also thoroughly examined in the past in a number of studies utilizing various *in vivo* models as well as in human subjects. Indeed, GA-based cream and serum formulations (2.5–3% GA content) have been safely applied for 4–6 weeks in a number of studies involving human subjects (Armanini et al., 2005; Silverberg, 2014; Grippaudo and Di Russo, 2016). Further, due to the inhibitory effects of GA on type 2 11β-hydroxysteroid dehydrogenase activity, plasma levels of aldosterone and cortisol were measured in human subjects receiving topical GA treatment for 1 month (Armanini et al., 2005). Essentially, neither effects on blood pressure, cortisol or aldosterone plasma levels nor renin activity were detected (Armanini et al., 2005).

In summary, the results of this study indicate that GA has the capacity to interfere with the activation of biomechanically stressed venous ECs and inhibits attenuated remodeling of superficial veins upon topical application. Although it remains to be investigated how GA exerts its effects, it is tempting to speculate that skin care products containing this bioactive compound may decelerate the formation of spider veins.

## Author contributions

HK, CA, and AW: Performed the experiments; MH: Contributed expertise and wrote the manuscript; HK, CS, CA, AW, and TK: Conceived the study, analyzed data and wrote the manuscript.

### Conflict of interest statement

The authors declare that the research was conducted in the absence of any commercial or financial relationships that could be construed as a potential conflict of interest.

## References

[B1] Akasaka Y. Yoshida T. Tsukahara M. Hatta A. Inoue H. (2011). Glycyrrhetinic acid prevents cutaneous scratching behavior in mice elicited by substance P or PAR-2 agonist. Eur. J. Pharmacol. 670, 175–179. 10.1016/j.ejphar.2011.08.043 21925497

[B2] Armanini D. Nacamulli D. Francini-Pesenti F. Battagin G. Ragazzi E. Fiore C. (2005). Glycyrrhetinic acid, the active principle of licorice, can reduce the thickness of subcutaneous thigh fat through topical application. Steroids 70, 538–542. 10.1016/j.steroids.2005.01.007 15894038

[B3] Atta H. M. (2012). Varicose veins: role of mechanotransduction of venous hypertension. Int. J. Vasc. Med. 2012:538627 10.1155/2012/538627 22489273 PMC3303599

[B4] Behringer E. J. Socha M. J. Polo-Parada L. Segal S. S. (2012). Electrical conduction along endothelial cell tubes from mouse feed arteries: confounding actions of glycyrrhetinic acid derivatives. Br. J. Pharmacol. 166, 774–787. 10.1111/j.1476-5381.2011.01814.x 22168386 PMC3417504

[B5] Chang Y. L. Chen C. L. Kuo C. L. Chen B. C. You J. S. (2010). Glycyrrhetinic acid inhibits ICAM-1 expression via blocking JNK and NF-kappaB pathways in TNF-alpha-activated endothelial cells. Acta Pharmacol. Sin. 31, 546–553. 10.1038/aps.2010.34 20418897 PMC4002749

[B6] Du W. Mills I. Sumpio B. E. (1995). Cyclic strain causes heterogeneous induction of transcription factors, AP-1, CRE binding protein and NF-kB, in endothelial cells: species and vascular bed diversity. J. Biomech. 28, 1485–1491. 10.1016/0021-9290(95)00096-8 8666588

[B7] Eklöf B. Rutherford R. B. Bergan J. J. Carpentier P. H. Gloviczki P. Kistner R. L.. (2004). Revision of the CEAP classification for chronic venous disorders: consensus statement. J. Vasc. Surg. 40, 1248–1252. 10.1016/j.jvs.2004.09.027 15622385

[B8] Eschrich J. Meyer R. Kuk H. Wagner A. H. Noppeney T. Debus S.. (2016). Varicose remodeling of veins is suppressed by 3-Hydroxy-3-methylglutaryl coenzyme a reductase inhibitors. J. Am. Heart Assoc. 5:e002405 10.1161/JAHA.115.002405 26908399 PMC4802467

[B9] Feldner A. Otto H. Rewerk S. Hecker M. Korff T. (2011). Experimental hypertension triggers varicosis-like maladaptive venous remodeling through activator protein-1. FASEB J. 25, 3613–3621. 10.1096/fj.11-185975 21685329

[B10] Grippaudo F. R. Di Russo P. P. (2016). Effects of topical application of B-Resorcinol and Glycyrrhetinic acid monotherapy and in combination with fractional CO_2_ laser treatment for benign hand hyperpigmentation treatment. J. Cosmet. Dermatol. 15, 413–419. 10.1111/jocd.12241 27325103

[B11] Heiss M. Hellström M. Kalén M. May T. Weber H. Hecker M.. (2015). Endothelial cell spheroids as a versatile tool to study angiogenesis *in vitro*. FASEB J. 29, 3076–3084. 10.1096/fj.14-267633 25857554

[B12] Hertel W. Peschel G. Ozegowski J. H. Muller P. J. (2006). Inhibitory effects of triterpenes and flavonoids on the enzymatic activity of hyaluronic acid-splitting enzymes. Arch. Pharm. 339, 313–318. 10.1002/ardp.200500216 16718670

[B13] Huang R. Y. Chu Y. L. Huang Q. C. Chen X. M. Jiang Z. B. Zhang X.. (2014). 18β-Glycyrrhetinic acid suppresses cell proliferation through inhibiting thromboxane synthase in non-small cell lung cancer. PLoS ONE 9:e93690. 10.1371/journal.pone.0093690 24695790 PMC3973544

[B14] Inoue H. Mori T. Shibata S. Koshihara Y. (1989). Modulation by glycyrrhetinic acid derivatives of TPA-induced mouse ear oedema. Br. J. Pharmacol. 96, 204–210. 10.1111/j.1476-5381.1989.tb11801.x 2924072 PMC1854326

[B15] Jayasooriya R. G. Dilshara M. G. Park S. R. Choi Y. H. Hyun J. W. Chang W. Y.. (2014). 18β-Glycyrrhetinic acid suppresses TNF-alpha induced matrix metalloproteinase-9 and vascular endothelial growth factor by suppressing the Akt-dependent NF-kappaB pathway. Toxicol. In Vitro 28, 751–758. 10.1016/j.tiv.2014.02.015 24613819

[B16] Kizub I. V. Lakhkar A. Dhagia V. Joshi S. R. Jiang H. Wolin M. S.. (2016). Involvement of gap junctions between smooth muscle cells in sustained hypoxic pulmonary vasoconstriction development: a potential role for 15-HETE and 20-HETE. Am. J. Physiol. Lung Cell. Mol. Physiol. 310, L772–L783. 10.1152/ajplung.00377.2015 26895643 PMC4836112

[B17] Kong S. Z. Chen H. M. Yu X. T. Zhang X. Feng X. X. Kang X. H.. (2015). The protective effect of 18β-Glycyrrhetinic acid against UV irradiation induced photoaging in mice. Exp. Gerontol. 61, 147–155. 10.1016/j.exger.2014.12.008 25498537

[B18] Kowalewski R. Sobolewski K. Wolanska M. Gacko M. (2004). Matrix metalloproteinases in the vein wall. Int. Angiol. 23, 164–169. 15507895

[B19] Krzesz R. Wagner A. H. Cattaruzza M. Hecker M. (1999). Cytokine-inducible CD40 gene expression in vascular smooth muscle cells is mediated by nuclear factor kappaB and signal transducer and activation of transcription-1. FEBS Lett. 453, 191–196. 10.1016/S0014-5793(99)00683-3 10403401

[B20] Kuk H. Arnold C. Meyer R. Hecker M. Korff T. (2017). Magnolol inhibits venous remodeling in mice. Sci. Rep. 7:17820. 10.1038/s41598-017-17910-0 29259201 PMC5736655

[B21] Matchkov V. V. Rahman A. Peng H. Nilsson H. Aalkjaer C. (2004). Junctional and nonjunctional effects of heptanol and glycyrrhetinic acid derivates in rat mesenteric small arteries. Br. J. Pharmacol. 142, 961–972 10.1038/sj.bjp.0705870 15210581 PMC1575116

[B22] Meissner M. H. (2005). Lower extremity venous anatomy. Semin. Intervent. Radiol. 22, 147–156. 10.1055/s-2005-921948 21326687 PMC3036282

[B23] Monder C. Stewart P. M. Lakshmi V. Valentino R. Burt D. Edwards C. R. (1989). Licorice inhibits corticosteroid 11 beta-dehydrogenase of rat kidney and liver: *in vivo* and *in vitro* studies. Endocrinology 125, 1046–1053. 10.1210/endo-125-2-1046 2752963

[B24] Nomura S. Yoshimura K. Akiyama N. Mikamo A. Furutani A. Aoki H.. (2005). HMG-CoA reductase inhibitors reduce matrix metalloproteinase-9 activity in human varicose veins. Eur. Surg. Res. 37, 370–378. 10.1159/000090339 16465063

[B25] North K. A. Sanders A. G. (1958). The development of collateral circulation in the mouse's ear. Circ. Res. 6, 721–727. 10.1161/01.RES.6.6.721 13585598

[B26] Partsch H. (2009). Varicose veins and chronic venous insufficiency. Vasa 38, 293–301. 10.1024/0301-1526.38.4.293 19998250

[B27] Pascarella L. Schmid-Schonbein G. W. Bergan J. (2005). An animal model of venous hypertension: the role of inflammation in venous valve failure. J. Vasc. Surg. 41, 303–311. 10.1016/j.jvs.2004.10.038 15768014

[B28] Pfisterer L. König G. Hecker M. Korff T. (2014a). Pathogenesis of varicose veins - lessons from biomechanics. Vasa 43, 88–99. 10.1024/0301-1526/a000335 24627315

[B29] Pfisterer L. Meyer R. Feldner A. Drews O. Hecker M. Korff T. (2014b). Bortezomib protects from varicose-like venous remodeling. FASEB J. 28, 3518–3527. 10.1096/fj.14-250464 24769668

[B30] Raffetto J. D. Khalil R. A. (2008). Mechanisms of varicose vein formation: valve dysfunction and wall dilation. Phlebology 23, 85–98. 10.1258/phleb.2007.007027 18453484

[B31] Raffetto J. D. Qiao X. Koledova V. V. Khalil R. A. (2008). Prolonged increases in vein wall tension increase matrix metalloproteinases and decrease constriction in rat vena cava: potential implications in varicose veins. J. Vasc. Surg. 48, 447–456. 10.1016/j.jvs.2008.03.004 18502086 PMC2575039

[B32] Sato H. Takino T. Okada Y. Cao J. Shinagawa A. Yamamoto E.. (1994). A matrix metalloproteinase expressed on the surface of invasive tumour cells. Nature 370, 61–65. 10.1038/370061a0 8015608

[B33] Silverberg J. I. (2014). Atopic dermatitis: an evidence-based treatment update. Am. J. Clin. Dermatol. 15, 149–164. 10.1007/s40257-014-0062-z 24464934

[B34] Sisto T. Reunanen A. Laurikka J. Impivaara O. Heliovaara M. Knekt P.. (1995). Prevalence and risk factors of varicose veins in lower extremities: mini-Finland health survey. Eur. J. Surg. 161, 405–414. 7548376

[B35] Smith P. C. (2015). Management of reticular veins and telangiectases. Phlebology 30(2 Suppl.), 46–52. 10.1177/0268355515592770 26556703

[B36] Sobieszczyk P. Borlaug B. A. Gornik H. L. Knauft W. D. Beckman J. A. (2010). Glycyrrhetinic acid attenuates vascular smooth muscle vasodilatory function in healthy humans. Clin. Sci. 119, 437–442. 10.1042/CS20100087 20515440

[B37] Somjen G. M. (1995). Anatomy of the superficial venous system. Dermatol. Surg. 21, 35–45. 10.1111/j.1524-4725.1995.tb00109.x 7600017

[B38] Tang Z. H. Zhang L. L. Li T. Lu J. H. Ma D. L. Leung C. H.. (2015). Glycyrrhetinic acid induces cytoprotective autophagy via the inositol-requiring enzyme 1alpha-c-Jun N-terminal kinase cascade in non-small cell lung cancer cells. Oncotarget 6, 43911–43926. 10.18632/oncotarget.6084 26549806 PMC4791276

[B39] Thomson L. (2016). Sclerotherapy of telangiectasias or spider veins in the lower limb: a review. J. Vasc. Nurs. 34, 61–62. 10.1016/j.jvn.2016.04.002 27210454

[B40] Um S. J. Park M. S. Park S. H. Han H. S. Kwon Y. J. Sin H. S. (2003). Synthesis of new glycyrrhetinic acid (GA) derivatives and their effects on tyrosinase activity. Bioorg. Med. Chem. 11, 5345–5352. 10.1016/j.bmc.2003.09.046 14642578

[B41] Wang Z. Y. Agarwal R. Zhou Z. C. Bickers D. R. Mukhtar H. (1991). Inhibition of mutagenicity in Salmonella typhimurium and skin tumor initiating and tumor promoting activities in SENCAR mice by glycyrrhetinic acid: comparison of 18 alpha- and 18 beta-stereoisomers. Carcinogenesis 12, 187–192. 10.1093/carcin/12.2.187 1899808

[B42] Woodside K. J. Hu M. Burke A. Murakami M. Pounds L. L. Killewich L. A.. (2003). Morphologic characteristics of varicose veins: possible role of metalloproteinases. J. Vasc. Surg. 38, 162–169. 10.1016/S0741-5214(03)00134-4 12844106

[B43] World Medical Association declaration of Helsinki (1997). Recommendations guiding physicians in biomedical research involving human subjects. JAMA 277, 925–926. 9062334

[B44] Zhao H. Hiroi T. Hansen B. S. Rade J. J. (2009). Cyclic stretch induces cyclooxygenase-2 gene expression in vascular endothelial cells via activation of nuclear factor kappa-beta. Biochem. Biophys. Res. Commun. 389, 599–601. 10.1016/j.bbrc.2009.09.028 19748489 PMC2763434

[B45] Zhu J. Chen M. Chen N. Ma A. Zhu C. Zhao R.. (2015). Glycyrrhetinic acid induces G1phase cell cycle arrest in human nonsmall cell lung cancer cells through endoplasmic reticulum stress pathway. Int. J. Oncol. 46, 981–988. 10.3892/ijo.2015.2819 25573651 PMC4324580

