# Supplementary material for: Glycyrrhetinic Acid Antagonizes Pressure-Induced Venous Remodeling in Mice
Source: Front Physiol. 2018 Apr 4;9:320. doi: 10.3389/fphys.2018.00320 (PMC5893715; doi:10.3389/fphys.2018.00320)
Supplement: Supplementary file 1 [file Presentation1.PDF]

Supplemental Figure S1

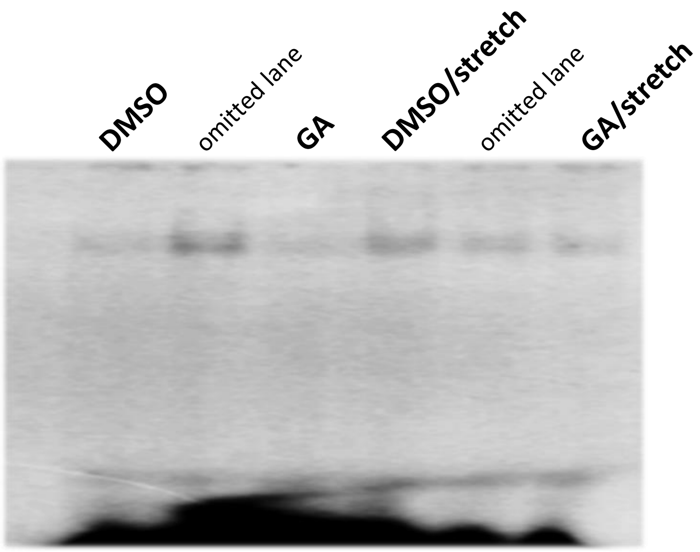

Figure S1. Uncropped version of the gel shown in Figure 4

HUVECs were treated with GA (40 µM) or DMSO control vehicle for 1.5 hours and subjected to biomechanical stretch (15% cyclic elongation at 0.5 Hz) for 6 hours. Binding of the mechanosensitive transcription factor AP-1 was assessed in an EMSA assay.

Supplemental Figure S2

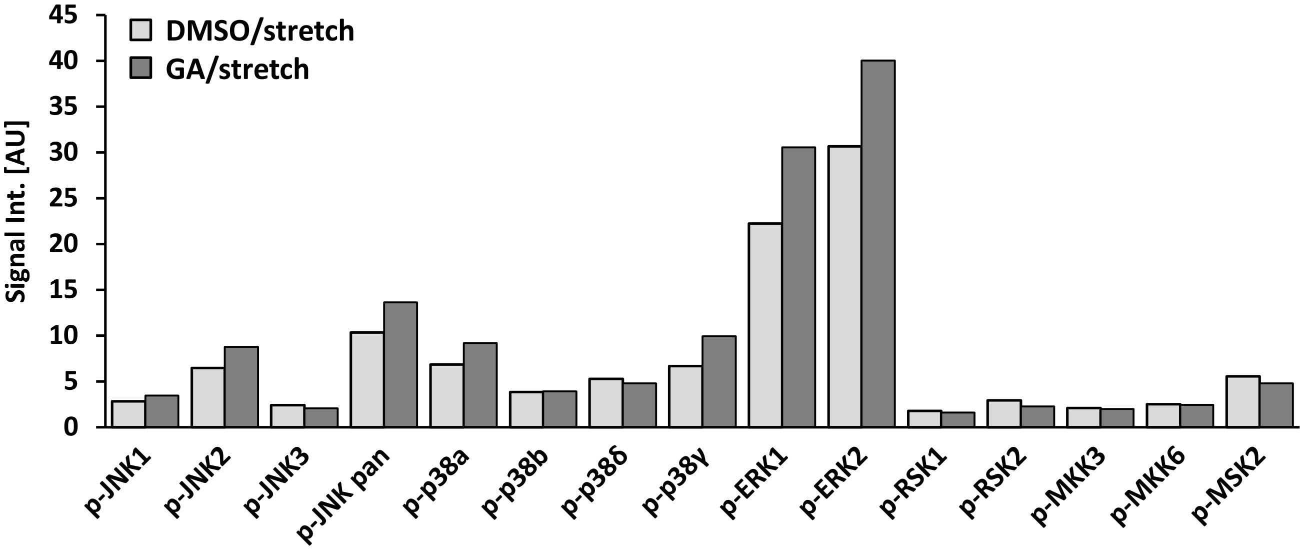

Figure S2: GA does not inhibit phosphorylation of MAP kinases in biomechanically stimulated HUVECs
HUVECs were treated with GA or DMSO for 1.5 hours and subjected to biomechanical stretch for 15 min. The phosphorylation level of several MAP kinases was assessed by applying the Proteome Profiler Array® technique (biotechne; immunoblot-based method utilizing phospho-target-specific antibodies to detect the phosphorylation level of proteins). GA treatment did not attenuate the phosphorylation level of MAP kinases in stretch-exposed HUVEC.

Supplemental Figure S3

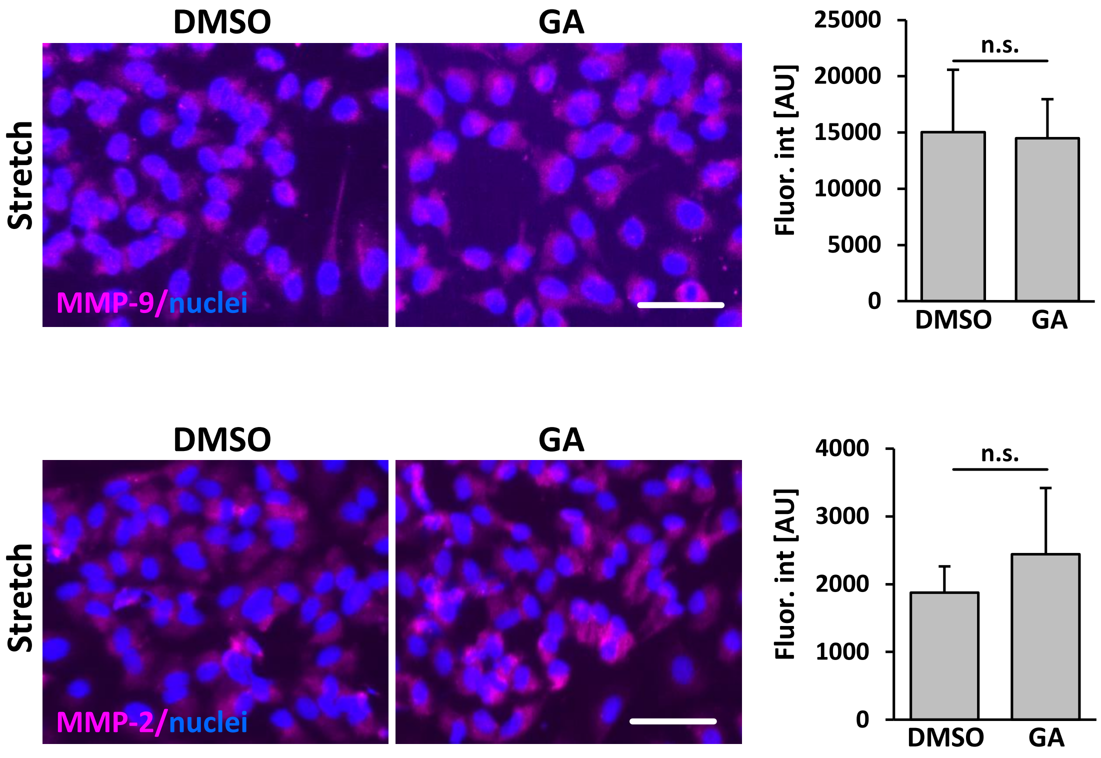

Figure S3. Glycyrrhetinic acid does not suppress MMP-2/9 levels in stretch-exposed HUVECs
HUVECs were exposed to biomechanical stretch (15% cyclic elongation at 0.5 Hz) for 24 hours with or without Glycyrrhetinic acid pretreatment (1.5 hours) followed by automated quantitative immunofluorescence analyses determining MMP-9/2-specific fluorescence intensity per cell. Bars represent the means ±SD of values obtained from 5 microscopic fields of view of 1 of 2 experiments with comparable results (n.s. = not significant; scale bars = 50 µm).

Supplemental Figure S4

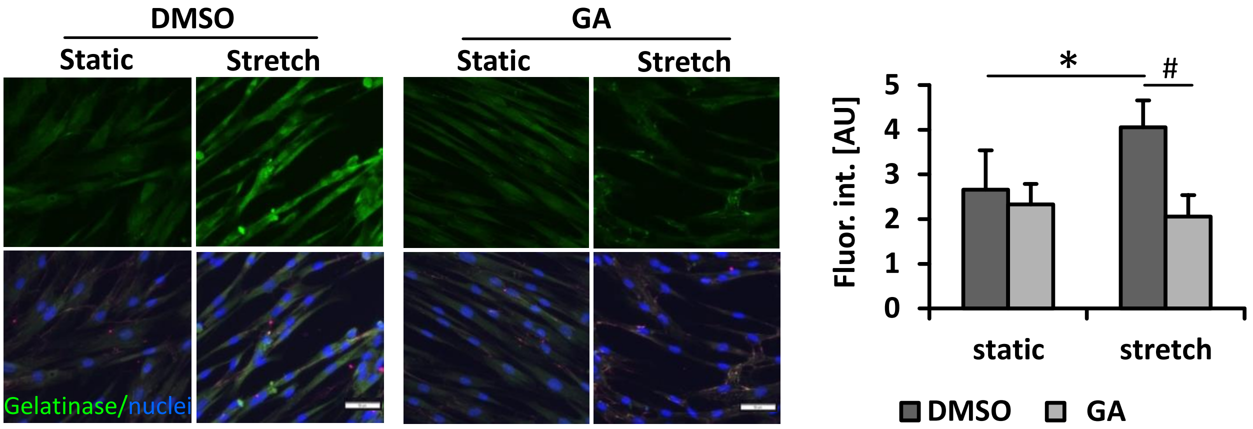

Figure S4. Glycyrrhetinic acid attenuates gelatinase activity in stretch-exposed HUVSMCs
HUVSMCs were exposed to biomechanical stretch (15% elongation at 0.5 Hz) for 24 hours with or without GA treatment followed by quantitative immunofluorescence detection indicating gelatinase activity (green fluorescence, *p<0.05, #p<0.05; bars represent the mean +/- SD of fluorescence values obtained from 5 microscopic fields of view; one representative experiment out of 5 with comparable results is shown; scale bar: 50 µm)

Supplemental Figure S5

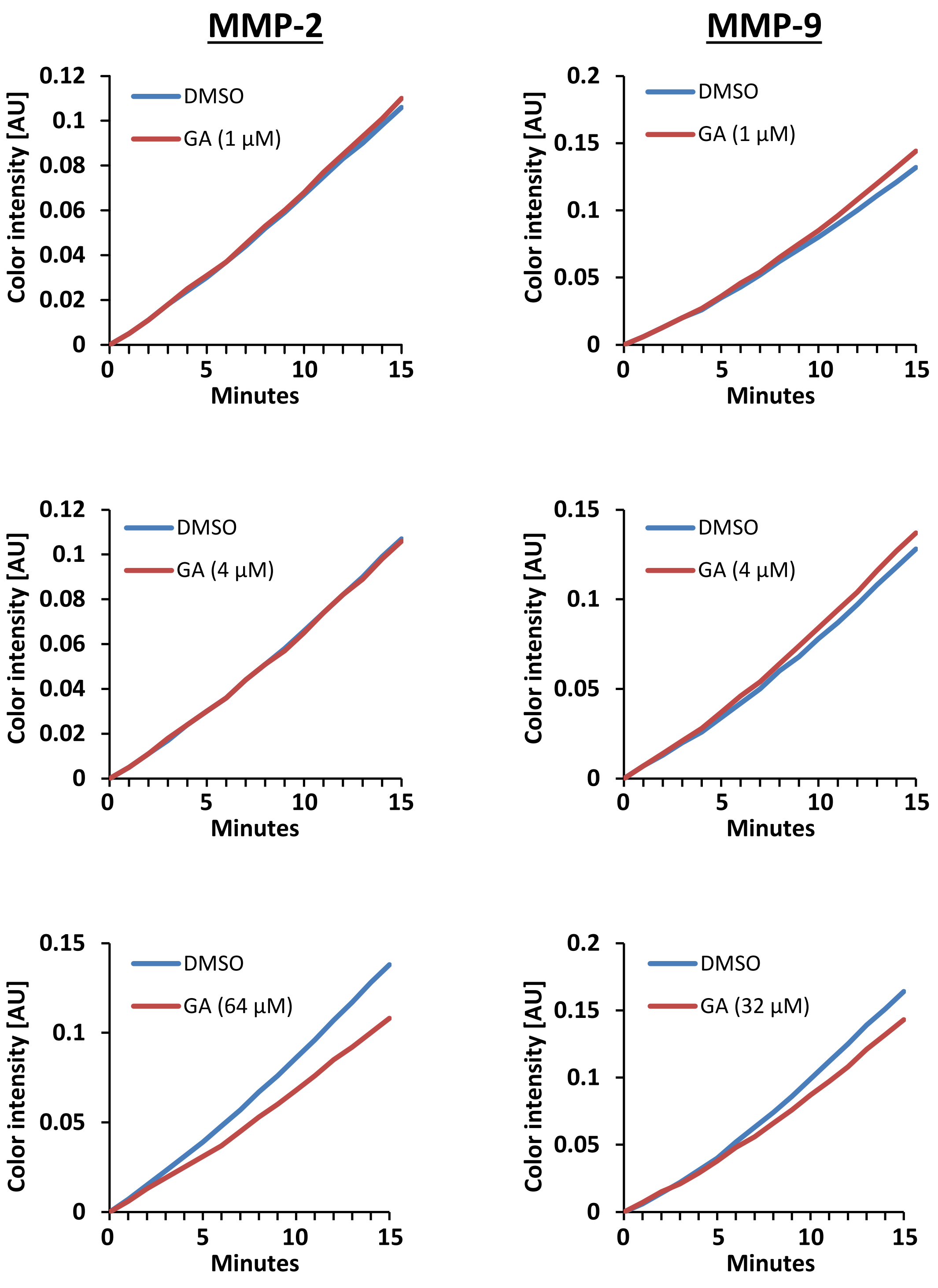

Figure S5. GA inhibits enzymatic activity of MMP-2

The capacity of GA to interfere with the activity of recombinant human MMP2 was assessed by applying a colorimetric enzyme activity assay. While lower concentrations showed no or minor effects, GA (64 µM) lowered the MMP2 activity as compared to the corresponding DMSO solvent control (lower left graph). Slight but not significant inhibitory effects of GA were also observed for MMP9 (right graphs).
